# Wogonin as a targeted therapeutic agent for EBV (+) lymphoma cells involved in LMP1/NF-κB/miR-155/PU.1 pathway

**DOI:** 10.1186/s12885-017-3145-4

**Published:** 2017-02-21

**Authors:** Xue Wu, Ping Liu, Haijun Zhang, Yuan Li, Jumah Masoud Mohammad Salmani, Fei Wang, Ke Yang, Rong Fu, Zhewei Chen, Baoan Chen

**Affiliations:** 1 Department of Hematology and Oncology (Key Department of Jiangsu Medicine), Medical School, the Affiliated Zhongda Hospital, Southeast University, Nanjing, 210009 China; 2 Department of Gastroenterology, Medical School, The Second Hospital of Nanjing Affiliated to Southeast University, Nanjing, China; 3 State Key Laboratory of Bioelectronics, School of Biological Science and Medical Engineering, Southeast University, Nanjing, China; 4 State Key Laboratory of Natural Medicines, Jiangsu Key Laboratory of Carcinogenesis and Intervention, China Pharmaceutical University, Nanjing, China

**Keywords:** Apoptosis, EBV infection, Lymphoma, NF-κB, Wogonin

## Abstract

**Background:**

Wogonin is an encouraging choice for clinical use owing to its potent anti-tumor and anti-inflammatory effects with the high safety profile. However, wogonin for targeted therapy of lymphoma was not well addressed. In this study, we focused on its anticancer effect alongside with the underlying mechanisms for targeted therapy in EBV-positive lymphoma. This will facilitate its introduction to clinical use, which is planned in the near future.

**Methods:**

Cell proliferation was studied by CCK8. Flow cytometry was used to analyze the apoptosis and the cycle arrest of cells. Further, we also used immunofluorescent staining to detect the morphologic changes of the apoptotic cells. The expression of LMP1/miR-155/p65/pp65/PU.1 was evaluated by quantitative real-time PCR (qRT-PCR) and western blot, while that of NF-κB was analyzed by EMSA. At last, immunohistochemical staining was applied to assess the expression of target proteins and relevant molecules.

**Results:**

In vitro, wogonin induced the apoptosis of Raji cells by downregulating the expression of NF-κB through LMP1/miR-155/NF-κB/PU.1 pathway, which was in a dose and time-dependent manner. In vivo, wogonin could suppress tumor growth, associated with the downregulation of ki67, p65 and upregulation of PU.1.

**Conclusions:**

Wogonin could suppress tumor growth and induce cell apoptosis by inhibiting the expression of NF-κB. Taken these findings, we concluded that wogonin could be a potential targeted therapeutic agent for EBV-positive lymphoma with the expression of LMP1 through the pathway of LMP1/NF-κB/miR-155/PU.1.

**Electronic supplementary material:**

The online version of this article (doi:10.1186/s12885-017-3145-4) contains supplementary material, which is available to authorized users.

## Background

In 2015, 80900 cases were newly diagnosed with lymphoma, accounting about 5% among all cases diagnosed with tumors. The mortality of lymphoma increased consistently during the last years [1]. Epstein-Barr virus (EBV), known as an oncogenic human herpes virus, is responsible for the pathogenesis of Burkitt’s lymphoma (BL), Hodgkin lymphoma (HL), extranodal NK/T cell lymphoma and parts of diffuse large B cell lymphoma (DLBCL) [2–4]. EBV-related oncogenesis is primarily associated with latency as well as some small noncoding RNAs, such as EBV-encoded small RNAs (EBERs) and microRNAs (miRs) [5, 6]. The expression of nuclear antigens and latent membrane proteins principally induce the proliferation of B cells [7, 8]. In addition, 25 viral pre-miRs are expressed during the latent infection of EBV. They regulate the expressions of the relevant cellular miRs such as miR-155, which has been recognized as a potential oncogene in activated B-cell (ABC) lymphomas, through the NF-κB pathway [6, 9].

Researches have indicated that the infection of EBV is an aggressive course for both elderly and young patients with lymphoma [10, 11]. Conventional CHOP regimens (Adriamycin, Vincristine, Cyclophosphamide, Prednisone) lead to a poor outcome with overall survival around 14 months [12]. For CD20-positive B-cell neoplasms, the addition of Rituximab can improve the outcomes of these patients [13], but in a small series of cases, patients with type III latency die within only 1 year even treated with Rituximab [14, 15]. Another promising novel therapy for EBV-positive B cell lymphoma is Bortezomib, a proteasome inhibitor, which has shown anti-tumor effect by inhibiting nuclear factor-κB (NF-κB) activity both in vitro and in vivo [16, 17]. Unfortunately, severe immunosuppression and myelosuppression are the main limitations preventing the wide clinical use of Rituximab and Bortezomib. Hence, new drugs with good tolerance and efficacy are highly demanded.

**Fig. 1:**
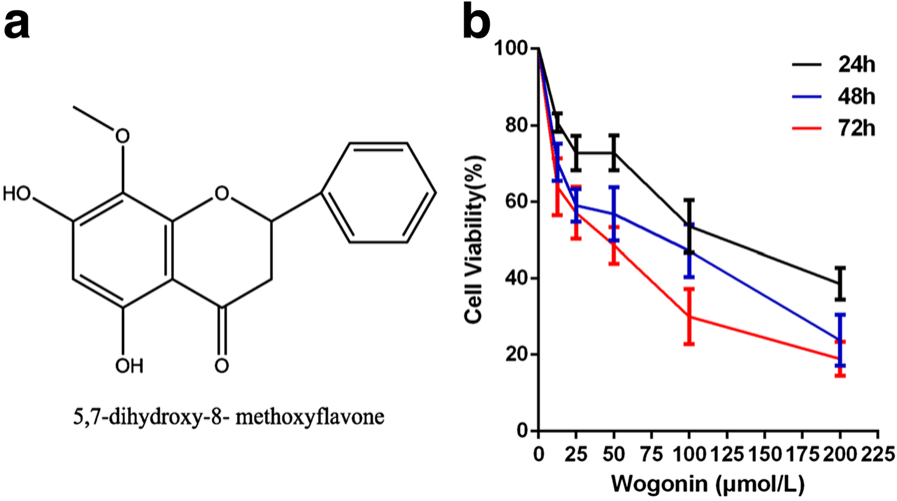
The inhibitory effect of Wogonin on Raji cells at different treatment times. **a** The chemical structure of Wogonin. Molecular formula: C_16_H_12_O_5_. Molecular weight: 284.26; **b** The inhibitory effect of Wogonin on Raji cells at different concentration after treated for 24h, 48h and 72h. Bars are the mean ± SD (*n* = 3)

During the past two decades, wogonin (5, 7-dihydroxy-8-methoxyflavone) has been identified as a potent apoptositic inducer for cancer cells with minor side effects [18, 19]. Wogonin is extracted from Scutellaria baicalensis Georgi (Huangqin), a perennial labiatae, and its molecular formula is C_16_H_12_O_5_ (Fig. 1a). Several studies have shown its inhibitory activity on tumor cells growth through intrinsic mitochondria-mediated and extrinsic receptor-mediated pathways [20, 21]. Furthermore, the inhabitation of NF-κB by wogonin also plays an important role in cell proliferation [22, 23]. Thereby, wogonin largely contributes to prevent the cellular immortalization and tumorigenesis.

Here, we assess the effect of wogonin on inducing the apoptosis of EBV (+) lymphoma cells and inhibiting tumor growth of xengrafted models. This involves the exploration of the possible molecular mechanisms and pathways through which wogonin exerts it action in this cell line.

## Methods

### Cell culture and Reagents

The human cell line Raji (ATCC® CCL-86™), a B lymphoma cell linewith EBV (+), was obtained from Shanghai Cell Bank of Chinese Academy of Sciences (Shanghai, China). The cells were cultured in RPMI-1640 medium (Gibco, Grand Island, NY, USA) supplemented with 10% heat-inactivated fetal bovine serum (Sijiqing, Hangzhou, China), 100 U/mL penicillin and 100μg/mL streptomycin (Sigma-Aldrich, St. Louis, MO, USA) in a 95% air and 5% CO_2_ atmosphere at 37 °C.

Wogonin (provided by the Key Laboratory of Carcinogenesis and Intervention, China Pharmaceutical University, China) was dissolved in dimethyl sulfoxide (DMSO, Sigma-Aldrich), stored at -20 °C, and diluted with medium when it was planned to be used in the experiment. MiR-155 inhibitor or miR-inhibitor normal control (NC) (GenePharma) was dissolved in DEPC-treated water prior to the experiment. Their sequence was as follows: miR-155 inhibitor (5’ to 3’) ACCCCUAUCACGAUUAGCAUUAA; miR-inhibitor NC (5’ to 3’) CAGUACUUUUGUGUAGUACAA. Lipofectamin2000 (GenePharma) was used for gene transfection of miR-155 inhibitor and NC according to its manufacturer’s protocols. PDTC (Beyotime, Nantong, China), used as the inhibitor of NF-κB, was dissolved in DMSO according to suggested concentration.

### Cytotoxicity assay

Cytotoxic effect of wogonin on the proliferating cells was detected by Cell Counting Kit 8 (CCK8, Dojindo, Kumamoto, Japan). Cells were seeded onto 96-well plates at a density of 3 × 10^4^ cells/well and treated with different concentrations of wogonin (0, 12.5, 25, 50, 100, 200 μmol/L) for 24, 48 and 72 h respectively. Then we added 10 μL of CCK8 solution into each well and incubated the cells for another 3 h. The absorbance was measured by Multiskan MK3 (Thermo Scientific, Shanghai, China) at 450 nm. After that, we calculated the cell viability as a percentage of the viable cells in the wogonin-treated group compared with the untreated control.

### Immunofluorescent staining

Cells were dripped on glass slides, fixed with 4% paraformaldehyde, for 30 min at room temperature. Then the cell nucleus was stained with DAPI (4, 6-diamidino-2-phenylindole) (Beyotime, Haimen, China) for 5 min and was photographed with a fluorescence confocal microscope (FV-1000, Olympus, Tokyo, Japan). Between each step, the cells were washed three times with PBS.

### Flow cytometry

Cells apoptotic rate was detected by Flow cytometry using Annexin V-FITC Apoptosis Detection Kit (Key-GEN, Nanjing, China) according to the manufacturer’s instructions. Two mL suspension of 10^5^ cells was stained with (Annexin-V-FITC and PI) kit solution in dark for 15 min. The assay then performed using FACSCalibur Flow Cytometry (BD, USA) at 488 nm.

The rate of cells cycle arrest was also detected by Flow cytometry using Cell Cycle Detection Kit (Key-GEN, Nanjing, China). Following its manufacturer’s instructions, 2 mL suspension of 10^6^ cells was fixed with 70% ethyl alcohol, and washed by PBS before staining. The assay performed in FACSCalibur Flow Cytometry (BD, USA) at 488 nm.

### RNA extraction and qRT-PCR assay

Total RNA was isolated from cells of each group using RNAiso Plus (TaKaRa, Dalian, China). Reverse transcription (RT) was performed with PrimeScript RT reagent kit with gDNA Eraser Kit (TaKaRa, Dalian, China), while real time PCR was carried out using FastStart Universal SYBR Green Master (ROX) Kit (Roche, Shanghai, China) according to manufacturer’s instructions on an ABI Prism 7500 HT device (Applied Biosystems). RT of total RNA of miR-155 and its cDNA synthesis were analyzed separately using different procedures. The RT contained: Total RNA 200ng, RT primer (10μmol/L) 0.2μL, 5 × Primescript buffer 4μL, Primescript RTase 0.5μL, dNTPs 2μL, Inhibitor (20U/μL) 0.5μL and DEPC-treated water to the total of 20μL. The program was 16 °C for 30min, 42 °C for 30min, 70 °C for 15min and 4 °C for 10min. The real time PCR contained: Premix EX Tag 10μL, hsa-mir155-5p F (10μmol/L) 0.5μL, Universal reverse primer (10μmol/L) 0.5μL, ROX II 0.4μL, Sybrgreen 1μL, cDNA 2μL and DEPC-treated water to the total of 20μL. The program was 95 °C for 30s, and then followed by 40 cycles of 95 °C for 5s and 60 °C for 34s.

The relative expression level of mRNAs was normalized to that of internal control U6 for miR-155, while GAPDH for other genes. The primer sequences were shown in Table 1.

**Table 1:** Sequence of each primer

Primer	Sequence
LMP1 (forward)	*5’-TGAGCAGGAGGGTGATCATC-3’*
LMP1 (reverse)	*5’-CTATTCCTTTGCTCTCATGC-3’*
PU.1 (forward)	*5’-CTCAGTCACCAGGTTTCC-3’*
PU.1 (reverse)	*5’-TCCAAGCCATCAGCTTCTC-3’*
Hsa-mir155-5p (forward)	*5-GCGGTTAATGCTAATCGTGAT-3*
Hsa-mir155-5p (reverse)	*5-GTGCAGGGTCCGAGGT-3*
GAPDH (forward)	*5’-CCATCACCATCTTCCAGGAG-3’*
GAPDH (reverse)	*5’-ACAGTCTTCTGGGTGGCAGT-3’*
U6 (forward)	*5’-CTCGCTTCGGCAGCACA-3’*
U6 (reverse)	*5’-AACGCTTCACGAATTTGCG-3’*

### Dual-luciferase reporter assay

Raji cells were seeded in 24-well plates at 5×10^5^/ml overnight. Then cells were transfected with 667ng of pNFκB-TA-luc reporter plasmid (Beyotime, Nanjing, China) and 133ng renilla luciferase-expressing plasmid as an internal control using Lipo2000 according to the manufacturer’s instructions for 24 h. After that, we set up three groups of cells, negative control group, positive control group (LPS was used as NF-κB activator) and test groups which were treated with wogonin at the concentration varying from 0μmol/L to 100μmol/L for 48 h before harvesting. Firefly luciferase and renilla luciferase activities were analyzed by Dual luciferase reporter assay kit (Beyotime, Nanjing, China). Renilla luciferase activities were used as an internal control.

### Electrophoresis mobility shift assay (EMSA)

Nuclears extracts from cells were performed with EMSA Detection Kit (Key-GEN, Nanjing, China) according to the manufacturer’s instructions. The NF-κB oligonucleotide comprised the sequence: 5’-AGCTATGTGGGTTTTCCCATGAGC-3’. To confirm the specificity of NF-κB, a 50-fold excess of NF-κB oligonucleotide, which was unlabeled, was added to the reaction mixture as a competitor. For EMSA, proteins were incubated with a NF-κB-specific ^32^P-labeled oligonucleotide and mix for binding. For supershift assay, antibodies were preincubated to the sample of interest prior to incubation with radiolabeled probe. The complexes formed were analyzed using Phosphor Imager Technology.

### Western blot analysis

Protein was extracted from cells and blots were incubated with diluted primary antibodies LMP1 (Abcam, UK), PU.1, p65 and phospho-p65 (Santa Cruz, USA) for overnight respectively at 4°C, and then incubated with horseradish peroxidase-conjugated goat anti-rabbit or mouse secondary antibody (Santa Cruz, USA) for 2h at room temperature. GAPDH (Santa Cruz, USA) was used as the internal control. For quantity, images were analyzed using Image J software (Bethesda, MD, USA).

### Mouse xenograft model

Four-week-old male BALB/c nude mice, 18–22 g, were purchased from Shanghai National Center for Laboratory Animals (Shanghai, China) and maintained in a pathogen-free environment. All the studies were performed in adherence with the Guidelines established by National Science Council, People of Republic of China. After the mice were injected with 1 × 10^7^ cells subcutaneously, tumor volume was measured every other day and calculated. The formula was V = a^2^b/2, in which “a” represented the smallest superficial diameter and “b” represented the largest superficial diameter. When the tumor volume reached nearly 50 mm^3^, the mice (*n* = 5/group) were randomly assigned into two groups: control group and wogonin-treated group (8mg ⁄kg per 2 days). Then the drug was administered via intraperitoneal injection every other day for 2 weeks. After 14 days, the mice were sacrificed and the tumors were removed and measured.

### Immunohistochemistry

UltraSensitive S-P IHC Kit (Maixin, Fuzhou, China) was used for immunohistochemical staining according to the manufacturer’s protocols. The sections were incubated with anti-p65, anti-PU.1 and anti-ki67 (1:100, Santa Cruz, USA) at 4 °C overnight. Then they were stained by a streptavidin-peroxidase system and the signal was visualized using diaminobenzidine substrate. Then the counterstaining with hematoxylin was performed to measure the microvessel density and the levels of PU.1 and ki67 by Image-Pro Plus 6.0 (Media Cybernetics, Silver Spring, MD, USA).

### Statistical analysis

All the values were shown as mean ± standard deviation (SD) from triplicate experiments performed in a parallel manner unless otherwise indicated. Data from the results was analyzed using an unpaired, two-tailed Student’s test. The level of significance was indicated as *P < 0.05 and **P < 0.01.

## Results

### Wogonin in vitro cytotoxicity study

In order to explore the potential anti-proliferative effects of wogonin on lymphoma cells, Raji was treated with wogonin at various concentrations (12.5–200 μmol/L) for 24 h, 48 h and 72 h respectively. A CCK8 assay was used to determine its proliferation, which showed that wogonin had the anti-proliferative effects on Raji cells in a dose- and time-dependent manner (Fig. 1b, Additional file 1: Table S1).

### Wogonin induces the apoptosis of Raji cells arrested at G1 phase

**Fig. 2:**
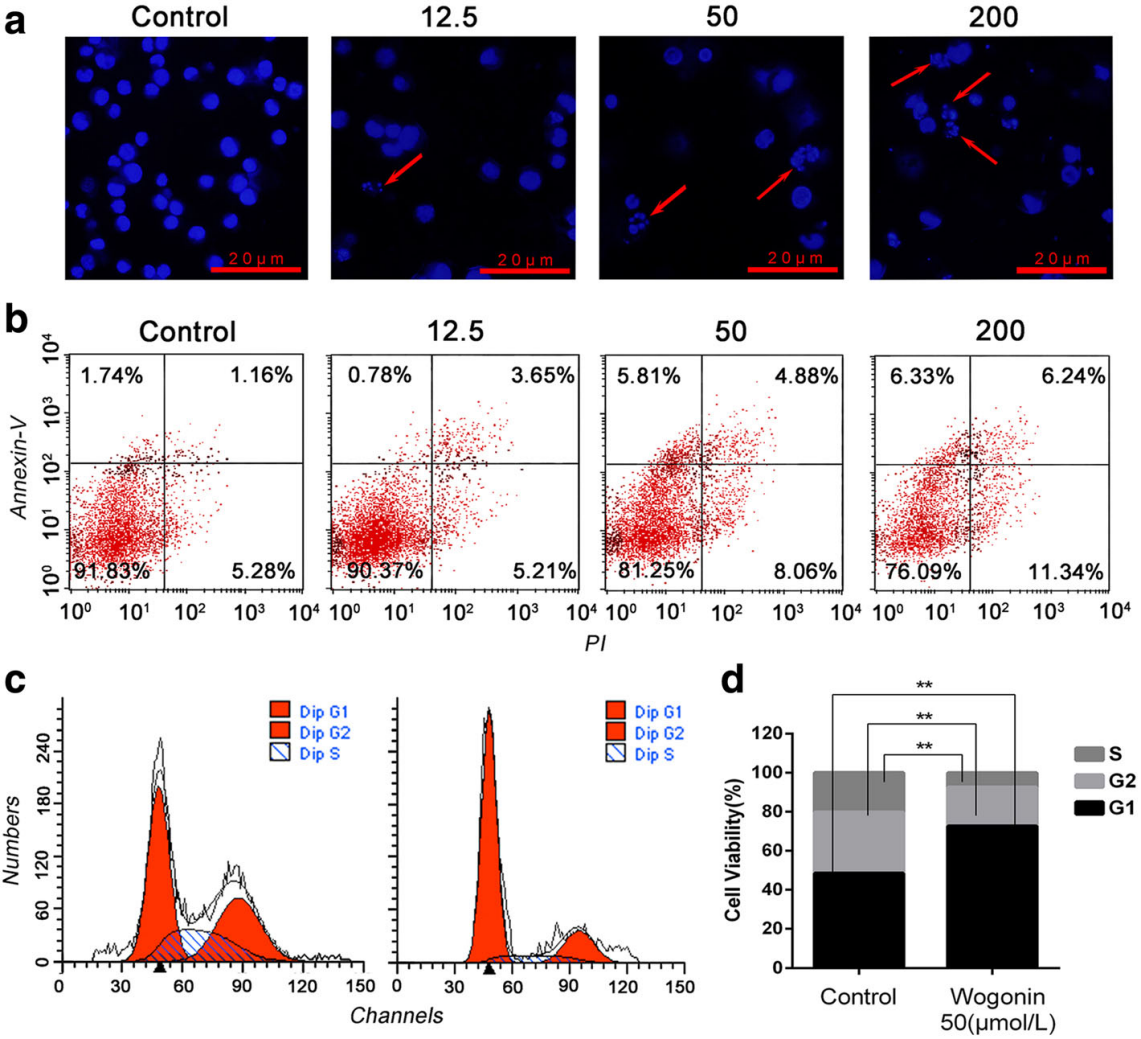
Wogonin induces the apoptosis of Raji cells arrested at G1 phase. **a** Fluorescence image of Raji cells treated with DAPI after 48 h incubation with wogonin at the concentration of 0 -200 μmol/L. Nuclear fragmentation and chromatin condensation are indicated with red arrows. **b** The apoptosis of Raji cells was determined by flow cytometry after incubation with wogonin at 0 -200 μmol/L for 48 h. The ratio of apoptosis was 6.44 ± 1.26%, 8.86 ± 0.74%, 12.94 ± 2.68% and 17.58 ± 3.73% for each group. **c** The cell cycle of Raji cells was arrested by wogonin (50 μmol/L) at G1 phase. **d** The ratio of S, G1, G2 phase was 22.77 ± 1.6%, 44.30 ± 1.91%, 32.93 ± 1.78% for the control group and 10.98 ± 1.17%, 70.04 ± 1.23%, 18.99 ± 0.65% for the wogonin treated group respectively, it was significantly up regulated at sub-G1 peak in wogonin treated group. The different levels of significance were indicated as *P < 0.05 and **P < 0.01

The morphological changes in the nucleolus caused by apoptosis of the cells were observed under a florescence microscope. Raji cells that were untreated with wogonin exhibited a pale blue florescence, whereas those treated with wogonin exhibited a nuclear fragmentation in the apoptotic cells at the concentration between 0 and 200 μmol/L of wogonin (Fig. 2a). The following Annexin V/PI double-staining assay also provided evidence that the percentage of apoptotic Raji cells increased after treated with wogonin from 12.5 μmol/L to 200 μmol/L in a dose-dependent manner (Fig. 2b). Furthermore, the Raji cells, which were incubated with 50μmol/L wogonin for 48 h were investigated by flow cytometry for cell cycle arrest (Fig. 2c). The difference between the percentage of S, G1 and G2 phase respectively in the control and wogonin-treated group was statically significant (Fig. 2d).

### MiR-155 plays a role in the apoptosis of Raji cells

**Fig. 3:**
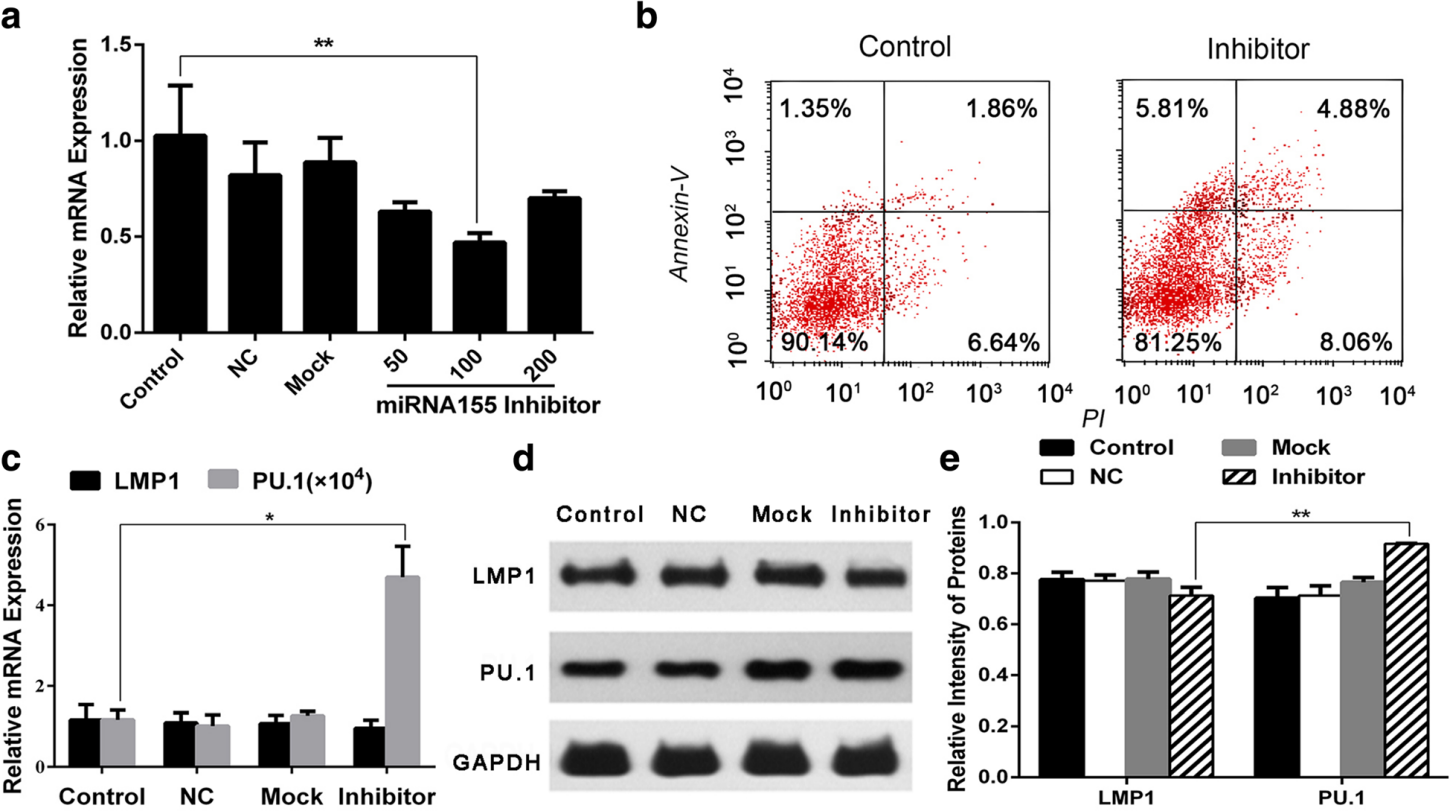
The expression of LMP1/miR-155/PU.1 in Raji cells with the process of miR-155 inhibitor treated apoptosis. **a** Effect of miR-155 inhibitor at the concentration of 50, 100 and 200 nmol/L for 48 h on the expression of miR-155 mRNA in Raji cells using PCR detection and analyzed by the ΔCt method, bars were shown as mean ± SD (*n* = 3) and represent miR-155/U6 ratios relative to the control group. The difference level of significance was (**P < 0.01). NC = Negative Control. Mock (cells + Lipo2000) = Blank Control. **b** Flow cytometry was used to detect the apoptosis of Raji cells after treated with miR-155 inhibitor at 100 nmol/L for 48 h. The ratio of apoptosis was 8.5 ± 0.74% and 12.94 ± 4.59% for the control and inhibitor-treated groups respectively. **c** Effect of miR-155 inhibitor at the concentration of 100 nmol/L for 48 h on LMP1 and PU.1 mRNA level of Raji cells detected by PCR and analyzed by the ΔCt method, bars were shown as mean ± SD (*n* = 3) and represent LMP1/GAPDH or PU.1/GAPDH ratios relative to the control group. The difference level of significance was (*P < 0.05). **d** Effect of miR-155 inhibitor 100nmol/L for 48 h on LMP1 and PU.1 protein expressions, the protein expression was analyzed by western blots; GAPDH was used as a loading control. **e** For quantity of (**d**), images were analyzed using Image J, bars were the mean ± SD (*n* = 3). The comparisons were made relative to control group, and the difference level of significance was (**P < 0.01)

To assess whether endogenous miR-155 played a role in regulating the proliferation of EBV (+) B lymphoma cells, Raji cells were transfected with either miR-155 inhibitor at suggested concentrations (0, 50, 100, 200nmol/L) or NC for miR-155 inhibitor from the illustration of the inhibitor using Lipo2000 for 48 h. qRT-PCR was used to evaluate the mRNA expression of miR-155 of each group. The results indicated that miR-155 inhibitor could down-regulate the expression of miR-155 in Raji cells, especially at the concentration of 100 nmol/L (Fig. 3a, Additional file 2: Table S2a). Meanwhile, when the expression of miR-155 was decreased, apoptosis started in Raji cells (Fig. 3b), which accounted for 12.94 ± 4.59% of the total and was significant compared with the control group.

Moreover, the expression of LMP1 and PU.1 in Raji cells were detected by qRT-PCR (Fig. 3c, Additional file 2: Table S2b and c) and Western blot (Fig. 3d and e). The results indicated that both of the transcription and translation of PU.1 were increased in the inhibitor treated group compared with the control group, while the expression of LMP1 had no difference between the two groups.

### Wogonin regulates the expression of miR-155 by NF-κB

**Fig. 4:**
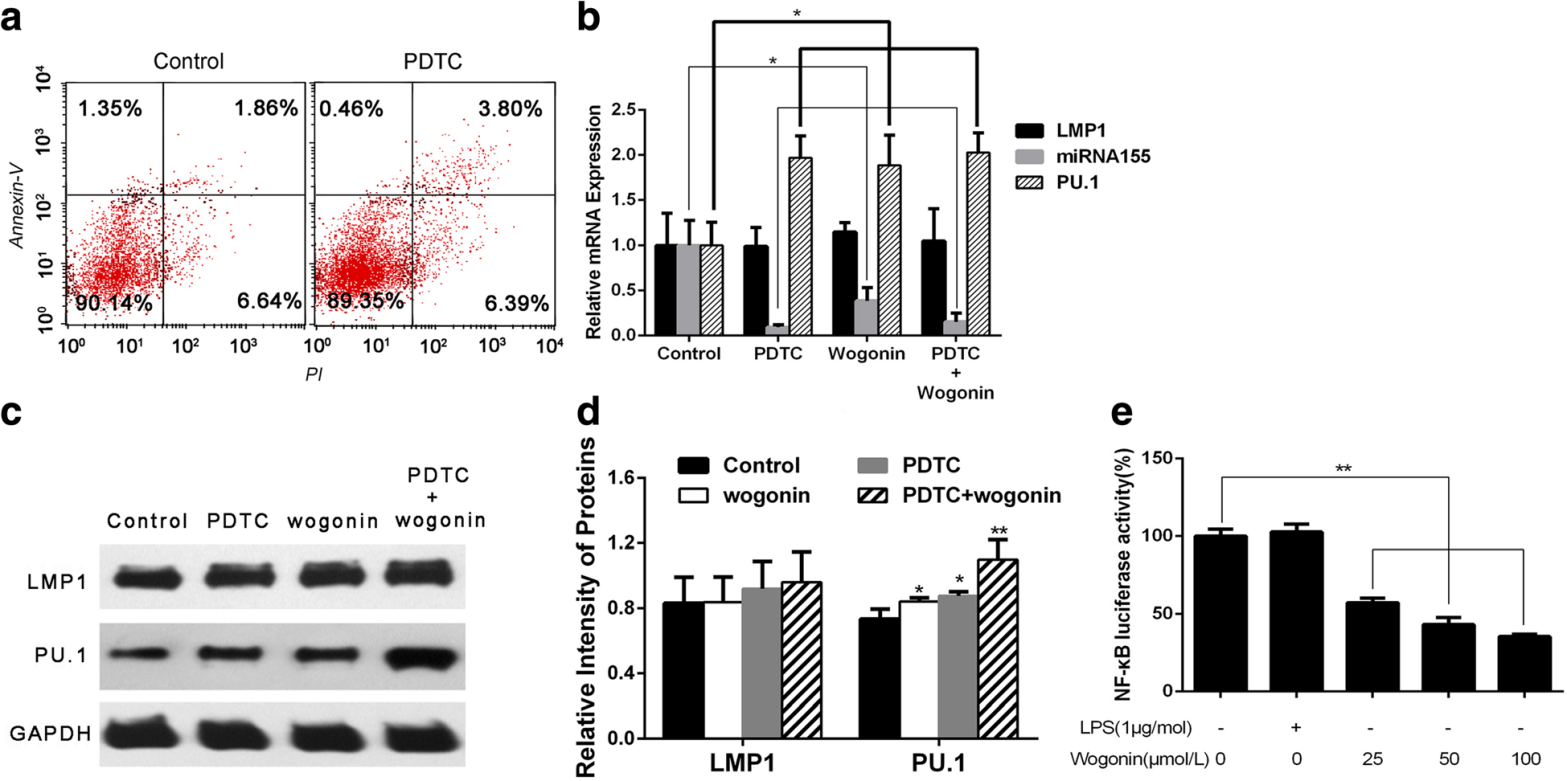
The influence of NF-κB inhibitor wogonin on LMP1/miR-155/PU.1 in Raji cells. **a** Flow cytometry was used to detect the apoptosis of Raji cells after treated with PDTC at 25μmol/L for 48 h. The ratio of apoptosis was 8.5 ± 0.74% and 10.19 ± 1.71% for the control and PDTC-treated groups respectively. **b** Effect of PDTC and PDTC with wogonin on the mRNA expression of LMP1, miR-155 and PU.1 of Raji cells detected by PCR and analyzed by the ΔCt method after treated at the concentration of 25 μmol/L PDTC, 50μmol/L wogonin and their combination for 48 h. Bars were shown as mean ± SD (*n* = 3) and represent LMP1/GAPDH or PU.1/GAPDH or miR-155/U6 ratios relative to the control group (*P < 0.05). **c** The protein expression of LMP1 and PU.1 were analyzed by western blots to evaluate the effect of PDTC (25 μmol/L), wogonin (50μmol/L) and their combination for 48 h in Raji cells. GAPDH was used as a loading control. **d** For quantity of (**c**), images were analyzed using Image J. Bars were the mean ± SD (*n* = 3). **e** Effect of wogonin on the NF-κB transcriptional activation was evaluated by Dual-luciferase reporter assay in wogonin-treated Raji cells. The comparisons were made relative to control group, and the difference levels of significance was indicated as (*P < 0.05 and **P < 0.01)

Firstly, we used NF-κB inhibitor (PDTC), to confirm whether or not the suppression of NF-κB could also induce the apoptosis of Raji cells. The results utilized by FCM were shown in Fig. 4a. Then the expressions of LMP1, miR-155 and PU.1 were evaluated using Real Time-qPCR in the control, PDTC, wogonin and PDTC + wogonin treated groups. Three important points could be concluded from the results: 1) although the expression of LMP1 did not show any difference among these groups, the expression of miR-155 was down-regulated significantly and on contrast the expression of PU.1 was increased after treated with PDTC or wogonin or their combination; 2) Wogonin could decrease the expression of miR-155 and increase the expression of PU.1 just like PDTC did; 3) the combination of the two drugs did not have an increased effect on these molecules compared with PDTC only (Fig. 4b, Additional file 3: Table S3). The protein level of LMP1 and PU.1 was evaluated by Western blot (Fig. 4c and d). However, these three points just indicated that wogonin could act as PDTC, because correlation did not mean causation, we did Dual-luciferase reporter assay to see whether wogonin could suppress the expression of NF-κB directly. As shown in Fig. 4e, wogonin inhibited NF-κB activity in Raji cells.

**Fig. 5:**
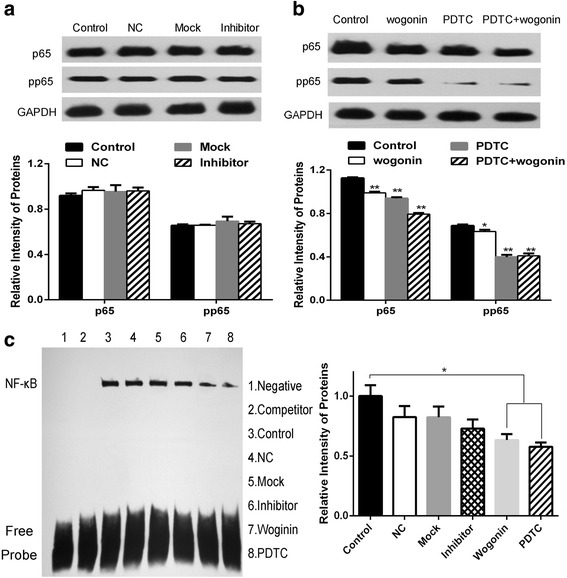
Wogonin down-regulates the protein expression of NF-κB. **a** Effect of miR-155 inhibitor on p65 and pp65 protein expression. Raji cells were treated with 100nmol/L inhibitor for 48 h. The protein expression was analyzed by western blots. GAPDH was used as a loading control. **b** Effect of wogonin, PDTC and wogonin + PDTC on p65 and pp65 protein expression. Raji cells were treated with 50μmol/L wogonin, 50μmol/L PDTC or their combination respectively for 48 h. The protein expression was analyzed by western blots. GAPDH was used as a loading control. **c** The effects of miR-155 inhibitor, wogonin and PDTC on NF-κB expression showed the clear suppressive effect of wogonin and PDTC, while miR-155 inhibitor had little effect on activation of NF-κB. Raji cells were incubated with 100nmol/L miR-155 inhibitor, 50μmol/L PDTC or 50μmol/L wogonin respectively for 48 h, and then analyzed for NF-κB expression by EMSA. The comparisons were made relative to control group using intensity of protein. All the images were detected by Image J. Bars were the mean ± SD(*n* = 3). The different levels of significance was indicated as (*P < 0.05 and **P < 0.01)

The effects of miR-155 inhibitor as well as the effect of wogonin, PDTC and their combination on p65 and pp65 protein expression were analyzed by western blot (Fig. 5a and b). For further confirmation about the suppression of wogonin on NF-κB, we separated Raji cells into eight groups and detected NF-κB by EMSA. As shown in Fig. 5c, wogonin, significantly suppressed the expression of NF-κB.

### Wogonin inhibits the growth of transplantable tumors and down-regulates protein level of p65 and PU.1

**Fig. 6:**
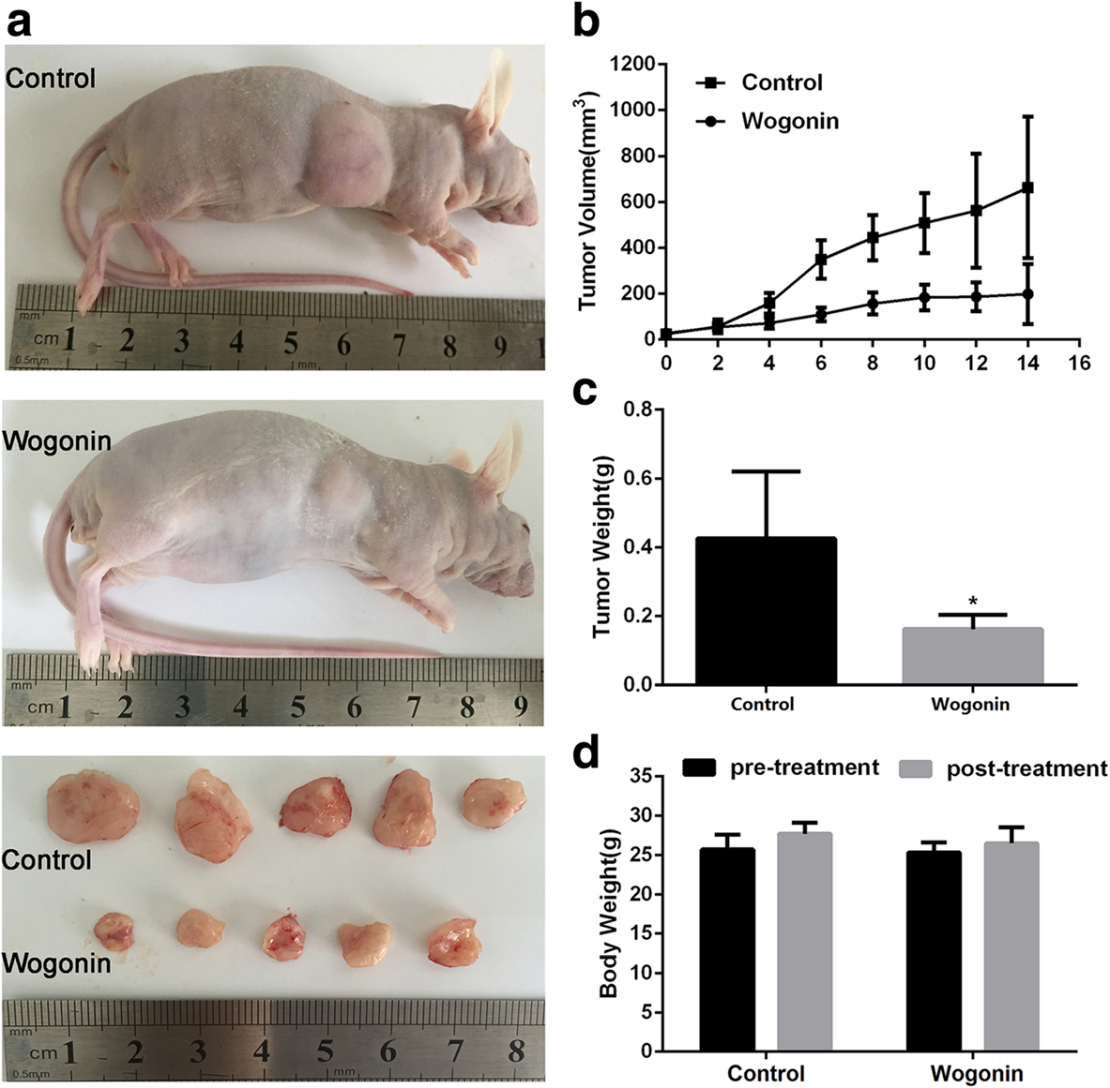
Wogonin inhibited tumor growth in a xenografts mouse model. **a** The BALB/c nude mice were injected with Raji cells for a few days followed by treatment with solvent or various doses of wogonin every other day for 14 days. Then, the mice were killed, tumor removed and photographed. **b**, **c**, **d** The tumor size, tumor weight and body weight measurements. Bars are shown as mean ± SD (*n* = 5). The comparisons were made relative to untreated controls, and the difference level of significance was indicated as (*P < 0.05)

Tumor xenografts transplanted by Raji cells were used to evaluate the anti-tumor effect of wogonin in BALB ⁄c nude mice in vivo. After intraperitoneal injection of wogonin every other day for 2 weeks, the tumors were moved and photographed (Fig. 6a). The average tumor size of control group was 663.4 ± 259.6 mm^3^, while that of wogonin treated groups was 199 ± 105.2 mm^3^ (Fig. 6b, Additional file 4: Table S4a). The average tumor weight of the control group was 0.426 ± 0.164 g, while that of wogonin treated groups was 0.162 ± 0.068 g (Fig. 6c, Additional file 4: Table S4b). The difference was obviously significant between the two groups.

The results indicated that wogonin had a dramatic effect on the inhibition of tumor growth. Moreover, wogonin treatment had very minor effects on the body weight of mice (Fig. 6d, Additional file 4: Table S4b), demonstrating that the maximal dose of wogonin (8 mg/kg per 2 days) had minimal toxic effects for mice.

**Fig. 7:**
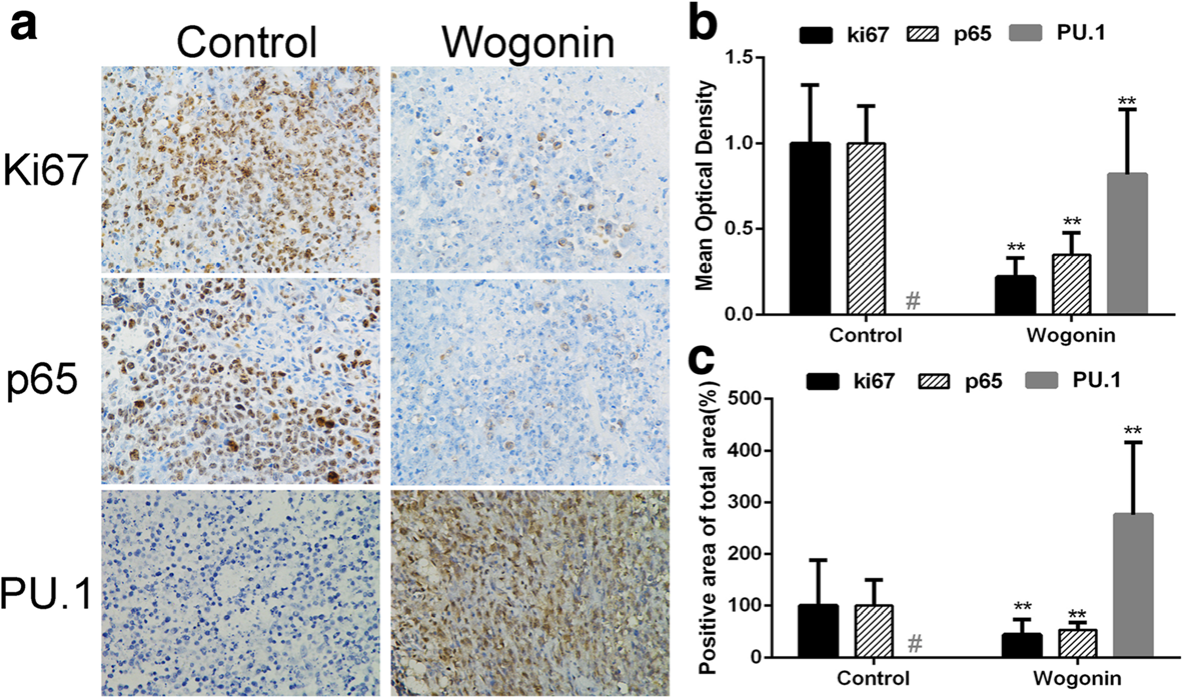
Wogonin inhibited tumor proliferation by regulating the expression of p65 and PU.1 in Raji xenografts mouse model. **a** Immunohistochemistry was performed in tumor sections with antibodies of ki-67, p65 and PU.1. The result showed a remarkable decrease in expression of ki-67 and p65, while an increase in expression of PU.1 in the wogonin treated groups with 8mg/kg compared with untreated control groups. **b**, **c** The images were quantified using Image Pro Plus. Mean optical densities and positive area of total area of ki-67, p65 and PU.1 were shown. Bars are the mean ± SD (*n* = 10). The comparisons were made relative to untreated controls (100% of control), and the difference level of significance was indicated as (**P < 0.01). # The expression of PU.1 had not been detected

To investigate the macroscopic observations and address the potential effect of wogonin in vivo, immunohistochemistry was performed. The results showed that ki67, the marker of tumor proliferation, was reduced by wogonin, as well as the component of NF-κB, p65. In addition, PU.1, a protein which had been recognized as the target of miR-155 which played an important role in cell apoptosis, was increased after wogonin treatment suggesting that wogonin could attenuate tumor growth (Fig. 7).

## Discussion

Since EBV (+) lymphoma is associated with poor prognosis [11], new therapeutic strategies are needed to improve the efficacy and reduce the toxicity of standard approaches. Potential new therapies include cellular immunotherapy, anti-viral treatments against EBV, monoclonal antibodies and inhibition of specific signaling targets [4, 24]. The cellular immunotherapy and anti-virus treatments belong to the approaches that target EBV-infected cells directly. The use of ex vivo-activated EBV-specific cytotoxic T cell lymphocytes (EBV-CTLs) and chimeric antigen receptor T-cells already had a certain curative effect on parts of EBV-related lymphomas [25–27]. However, the complex technologies and high costs limit the use of these therapies, despite of their very promising efficacy. On the other hand, EBV (+) lymphoma is not sensitive to anti-viral therapies because of lacking of EBV thymidine kinase, which is required for anti-viral activity. Therefore, histone deacetylase inhibitor, which is able to induce the lytic phase in EBV-infected lymphocytes, is usually used with Ganciclovir to improve the treatment of such disorders [28, 29]. Besides the use of anti-CD20 antibodies such as Rituximab, Brentuximab Vedotin is now used in relapsed or refractory lymphomas as an anti-CD30 antibody [30–33]. Notably, these monoclonal antibodies have a high safety profile in patients with immunodeficiency. Furthermore, suppression of the specific signaling pathways seems to be a conventional but always effective method to prevent pathological cells from proliferating malignantly. Scientists are constantly devoting themselves to discover new inhibitors. Currently, programmed cell death ligands attract lots of attention as an immune checkpoint blockage [34]. Meanwhile, traditional Chinese medicines (represented by wogonin) are trying to establish their own position as an anti-tumor reagent in a new era.

**Fig. 8:**
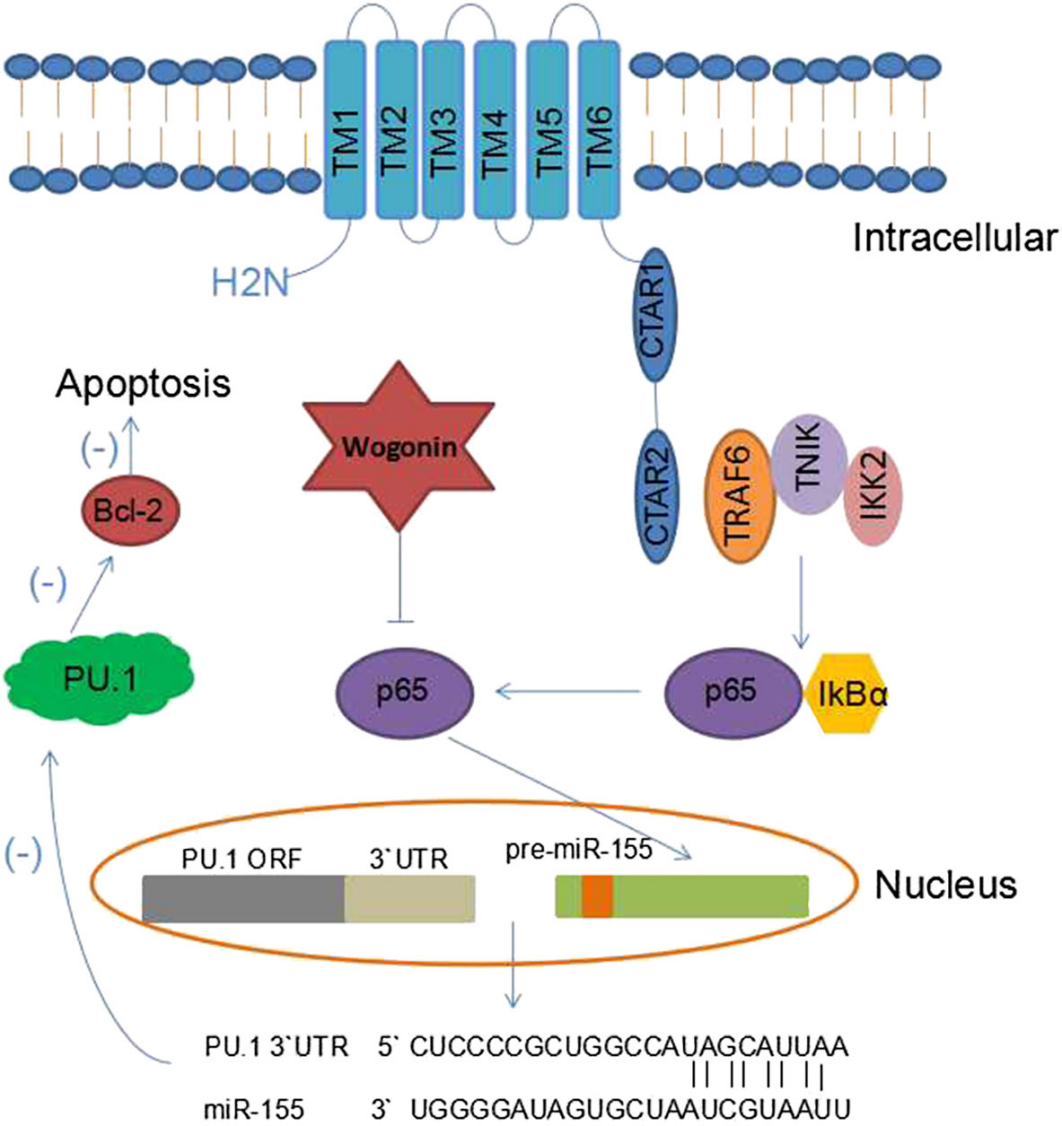
The molecular mechanism of wogonin on LMP1/NF-κB/miRNA-155/PU.1 pathway. LMP1 protein is consisted of TM1-6, CTAR1-2 and -H_2_N and activates IkBα by TRAF6. P65, which is binding to IkBα, is then free from IkBα and goes into the nucleus to bind on miRNA-155 promoter, inducing over-expression of miR-155. MiR-155 prevents the transcription of PU.1, and then suppresses the apoptosis of B lymphoma cells. Wogonin inhibited the pathway by suppressing p65

The current studies are relevant to explore a drug with high efficacy and low toxicity for the treatment of EBV (+) lymphoma, such as BL and EBV (+) DLBCL who usually have latency III type of EBV infection and high expressions of LMP1. LMP1 is consisted of six transmembrane proteins that are able to activate the two cytoplasmic signaling molecules namely C-terminal activator regions1 and 2 (CTAR1 and 2) constitutively to transform the properties from the extracellular region to the intracellular region [35, 36]. Then, the signaling domains go through tumor necrosis factor receptor (TNFR)-associated factors (TRAFs), especially TNFR6, to activate NF-κB, making p65 free from NF-κB complex [37, 38]. After p65 binding on the miR-155 promoter in the nucleus, miR-155 begin to be vastly produced [6, 39], leading to the down-expression of its target, which is named as transcription factor PU.1, and reducing the suppressive effect of PU.1 to the apoptotic relevant protein Bcl-2 (Fig. 8) [40, 41]. As this pathway has the capacity to keep B cells immortal, the inhibition of any molecule involved in this pathway will contribute to induce apoptosis of these pathological cells. Interestingly, in our studies, the results appeared to show a close relationship between LMP1 (+) cells and wogonin. Further exploration provided the evidence that miR-155 expressed highly in Raji cells, which could be down-regulated by wogonin. Thus, it was concluded that the LMP1 and miR-155 might have potential functions in LMP1 (+) lymphoma. In fact, some studies have focused on the role of wogonin on NF-κB in the process of inflammation [42, 43]. To access whether NF-κB could be inhibited by wogonin correlated with the suppression of miR-155, we compared the expression of NF-κB, p65, pp65 and miR-155 of wogonin-treated group with control group and access the effect of wogonin on NF-κB activity to confirm that wogonin down-regulated miR-155 by modulating NF-κB. Although it is not the first time to report the effect of wogonin on NF-κB, it has not been reported elsewhere that wogonin can induce the apoptosis of EBV (+) lymphoma cells by LMP1/NF-κB/miR-155/PU.1 signal pathway, suggesting wogonin as a promising drug to be used in the clinic for LMP1 (+) lymphoma in the future.

## Conclusions

In summary, we conclude that wogonin down-regulates the expression of NF-κB in Raji cells. The inhibition of NF-κB is associated with LMP1/NF-κB/miR-155/PU.1 pathway, suggesting the ability of wogonin to suppress the growth and induce the apoptosis of human LMP1 (+) lymphomas. Taken together, it was not only evidenced that wogonin could be a novel potential drug for LMP1 (+) lymphoma, but also implied that the detection of LMP1 should be considered for diagnosis. Therefore, our further studies will focus on the anti-tumor effect of wogonin on more EBV (+) lymphoma cell lines with LMP1 or LMP2 to identify the role of wogonin on EBV (+) lymphoma. In addition, the prognosis of patients with LMP1 (+) or (-) should be analyzed for the preparation of its use as a marker of therapy in clinics.

## Additional files

Additional file 1: Table S1. The inhibitory effect of Wogonin on Raji cells at different treatment times which is shown in Fig. 1. (DOC 35 kb)
Additional file 2: Table S2. Ct values of gene expression assessed by quantitative PCR in Raji cells after inhibition of miR-155. (DOC 25 kb)
Additional file 3: Table S3. Ct values of gene expression assessed by quantitative PCR in Raji cells after inhibition of NF-κB. (DOC 21 kb)
Additional file 4: Table S4. The volume, body weight and tumor weight of mouse. (DOC 38 kb)

## Abbreviations

ABC: Activated B-cell
BL: Burkitt’s lymphoma
CTAR: C-terminal activator regions
DLBCL: Diffuse large B cell lymphoma
EBERs: EBV-encoded small RNAs
EBV: Epstein-Barr virus
EBV-CTLs: EBV-specific cytotoxic T cell lymphocytes
EMSA: Electrophoresis mobility shift assay
HL: Hodgkin lymphoma
miRs: microRNAs
NC: Negative control
NF-κB: Nuclear factor-κB
qRT-PCR: Quantitative real-time PCR
RT: Reverse transcription
TNFR: Tumor necrosis factor receptor
