# Supplementary material for: Wogonin as a targeted therapeutic agent for EBV (+) lymphoma cells involved in LMP1/NF-κB/miR-155/PU.1 pathway
Source: BMC Cancer. 2017 Feb 21;17:147. doi: 10.1186/s12885-017-3145-4 (PMC5320633; doi:10.1186/s12885-017-3145-4)
Supplement: Additional file 1: Table S1. — The inhibitory effect of Wogonin on Raji cells at different treatment times which is shown in Fig. 1. (DOC 35 kb) [file 12885_2017_3145_MOESM1_ESM.doc]

Table S1 The inhibitory effect of Wogonin on Raji cells at different treatment times which is shown in Figure 1.

24 hours

| Concentration(μmol/L) | Test 1 (%) | Test 2(%) | Teat 3(%) |
| --- | --- | --- | --- |
| 0 | 100 | 100 | 100 |
| 12.5 | 80.14773 | 83.44352 | 78.67463 |
| 25 | 72.73552 | 77.34634 | 68.24634 |
| 50 | 72.89102 | 77.32653 | 68.24663 |
| 100 | 52.85733 | 47.11143 | 60.74754 |
| 200 | 39.96371 | 33.98425 | 41.86879 |

48 hous

| Concentration(μmol/L) | Test 1(%) | Test 2(%) | Teat 3(%) |
| --- | --- | --- | --- |
| 0 | 100 | 100 | 100 |
| 12.5 | 75.78661 | 66.37356 | 69.14646 |
| 25 | 61.65967 | 54.13678 | 61.35678 |
| 50 | 60.46431 | 48.75775 | 61.16744 |
| 100 | 46.89059 | 40.48936 | 54.16854 |
| 200 | 22.63275 | 17.78652 | 30.97943 |

72 hours

| Concentration(μmol/L) | Test 1(%) | Test 2(%) | Teat 3(%) |
| --- | --- | --- | --- |
| 0 | 100 | 100 | 100 |
| 12.5 | 67.19524 | 55.37835 | 69.18974 |
| 25 | 60.88536 | 49.22356 | 61.15732 |
| 50 | 51.69475 | 43.17874 | 50.97244 |
| 100 | 36.76908 | 30.66796 | 22.42326 |
| 200 | 17.9782 | 14.94745 | 23.71435 |
