# Supplementary material for: Wogonin as a targeted therapeutic agent for EBV (+) lymphoma cells involved in LMP1/NF-κB/miR-155/PU.1 pathway
Source: BMC Cancer. 2017 Feb 21;17:147. doi: 10.1186/s12885-017-3145-4 (PMC5320633; doi:10.1186/s12885-017-3145-4)
Supplement: Additional file 2: Table S2. — Ct values of gene expression assessed by quantitative PCR in Raji cells after inhibition of miR-155. (DOC 25 kb) [file 12885_2017_3145_MOESM2_ESM.doc]

Table S2

Ct values of gene expression assessed by quantitative PCR in Raji cells. ΔΔCt= ΔTest (target gene Ct - internal control Ct) - ΔControl (target gene Ct – internal control Ct).

a. MiRNA-155 expression

| Group | ΔCt | ΔΔCt |
| --- | --- | --- |
| Control | 5.409117222 |  |
| NC | 6.069059372 | 0.65994215 |
| Mock | 5.835310936 | 0.426193714 |
| RNAi 50 | 6.186961651 | 0.777844429 |
| RNAi 100 | 6.365642548 | 0.956525326 |
| RNAi 200 | 5.988800049 | 0.579682827 |

b. LMP1 expression

| Group | ΔCt | ΔΔCt |
| --- | --- | --- |
| Control | 8.67096742 |  |
| NC | 8.56609281 | -0.10487461 |
| Mock | 8.192087173 | -0.478880246 |
| RNAi 100 | 9.532596588 | 0.171104431 |

c. PU.1 expression

| Group | ΔCt | ΔΔCt |
| --- | --- | --- |
| Control | 17.51870251 |  |
| NC | 16.9847496 | -0.533952907 |
| Mock | 17.7987634 | 0.280060893 |
| RNAi 100 | 15.38578574 | -2.132916767 |
