# Supplementary material for: Wogonin as a targeted therapeutic agent for EBV (+) lymphoma cells involved in LMP1/NF-κB/miR-155/PU.1 pathway
Source: BMC Cancer. 2017 Feb 21;17:147. doi: 10.1186/s12885-017-3145-4 (PMC5320633; doi:10.1186/s12885-017-3145-4)
Supplement: Additional file 3: Table S3. — Ct values of gene expression assessed by quantitative PCR in Raji cells after inhibition of NF-κB. (DOC 21 kb) [file 12885_2017_3145_MOESM3_ESM.doc]

Table S3

Ct values of gene expression assessed by quantitative PCR in Raji cells. ΔΔCt= ΔTest (target gene Ct - internal control Ct) - ΔControl (target gene Ct – internal control Ct).

| Group | ΔCt | | | ΔΔCt | | |
| --- | --- | --- | --- | --- | --- | --- |
|  | LMP1 | MiR-155 | PU.1 | LMP1 | MiR-155 | PU.1 |
| Control | 9.361492157 | 9.37676254 | 20.10947446 |  | 0.001504226 | 8.83985E-07 |
| PDTC | 7.257255077 | 13.22374845 | 19.42840016 | -2.10423708 | 0.000104533 | 1.41732E-06 |
| Wogonin | 9.033487797 | 10.92364735 | 19.45431712 | -0.32800436 | 0.000514819 | 1.39209E-06 |
| PDTC+Wogonin | 9.639647007 | 12.87847454 | 19.30983927 | 0.27815485 | 0.000132798 | 1.53872E-06 |
