# Supplementary material for: Wogonin as a targeted therapeutic agent for EBV (+) lymphoma cells involved in LMP1/NF-κB/miR-155/PU.1 pathway
Source: BMC Cancer. 2017 Feb 21;17:147. doi: 10.1186/s12885-017-3145-4 (PMC5320633; doi:10.1186/s12885-017-3145-4)
Supplement: Additional file 4: Table S4. — The volume, body weight and tumor weight of mouse. (DOC 38 kb) [file 12885_2017_3145_MOESM4_ESM.doc]

Table S4

a. The volume of each tumor on xenografts after being received wogonin for 14 days.

Legth*width (mm)

Group of test

| No. | DAY 0 | DAY 2 | DAY 4 | DAY 6 | DAY 8 | DAY 10 | DAY 12 | DAY 14 |
| --- | --- | --- | --- | --- | --- | --- | --- | --- |
| 1 | 5.1*4.6 | 6.0*4.4 | 6.4*5.9 | 7.1*6.0 | 7.9*6.6 | 8.2*7.2 | 9.0*8.0 | 10.1*9.4 |
| 2 | 6.3*3.2 | 6.1*3.5 | 6.0*4.0 | 6.7*5.0 | 7.0*7.0 | 7.5*7.0 | 7.7*7.2 | 6.8*6.9 |
| 3 | 5.3*4.2 | 5.8*5.1 | 5.8*5.2 | 6.8*5.9 | 8.0*6.2 | 8.6*6.8 | 8.1*6.6 | 8.2*8.0 |
| 4 | 3.3*3.9 | 5.3*5.0 | 5.5*5.1 | 8.2*6.0 | 8.6*7.0 | 9.4*7.1 | 7.4*6.3 | 7.6*6.8 |
| 5 | 4.8*3.0 | 6.9*3.5 | 7.1*4.0 | 7.5*4.5 | 7.5*4.6 | 7.8*4.8 | 6.7*6.1 | 8.0*5.1 |

Group of control

| No. | DAY 0 | DAY 2 | DAY 4 | DAY 6 | DAY 8 | DAY 10 | DAY 12 | DAY 14 |
| --- | --- | --- | --- | --- | --- | --- | --- | --- |
| 1 | 4.0*3.5 | 5.0*5.0 | 8.0*5.6 | 12.1*8.0 | 12.7*9.0 | 13.0*9.2 | 14.0*10.2 | 9.3*8.2 |
| 2 | 3.5*3.5 | 5.2*4.1 | 7.6*7.0 | 11.3*9.2 | 11.0*9.0 | 11.7*9.3 | 9.0*7.7 | 12.1*7.8 |
| 3 | 4.3*3.0 | 4.6*4.1 | 8.9*5.0 | 12.4*6.6 | 13.1*7.3 | 12.9*7.3 | 11.9*7.5 | 13.4*11.7 |
| 4 | 3.5*3 | 4.4*4.0 | 9.0*7.0 | 11.1*8.1 | 12.0*8.0 | 12.9*8.3 | 14.5*9.5 | 13.5*12.1 |
| 5 | 5.3*4.1 | 6.0*6.0 | 7.0*6.6 | 11.0*8.0 | 12.5*9.5 | 13.8*10.0 | 12.6*11.5 | 15.0*10.1 |

b. The weight of each tumor and mouse before and after being received wogonin for 14 days.

Weight (g)

| No. | Group of teat | | | Group of control | | |
| --- | --- | --- | --- | --- | --- | --- |
|  | Tumor weight | Body weight | | Tumor weight | Body weight | |
|  |  | Before treated | After treated |  | Before treated | After treated |
| 1 | 0.17 | 27 | 27.5 | 0.5731 | 27 | 28 |
| 2 | 0.153 | 26 | 29.5 | 0.6896 | 27.5 | 26 |
| 3 | 0.11 | 25.2 | 26 | 0.3489 | 24.5 | 29 |
| 4 | 0.155 | 24 | 25 | 0.2582 | 26.5 | 26.5 |
| 5 | 0.224 | 24 | 24.5 | 0.2608 | 23 | 29 |
